# COGA phenotypes and linkages on chromosome 2

**DOI:** 10.1186/1471-2156-6-S1-S125

**Published:** 2005-12-30

**Authors:** Howard W Wiener, Rodney CP Go, Hemant Tiwari, Varghese George, Grier P Page

**Affiliations:** 1 Department of Epidemiology, School of Public Health, University of Alabama at Birmingham, Birmingham, AL, USA; 2 Department of Biostatistics, School of Public Health, University of Alabama at Birmingham, Birmingham, AL, USA; 3 Department of Biostatistics, Medical College of Georgia, Augusta, GA, USA

## Abstract

An initial linkage analysis of the alcoholism phenotype as defined by DSM-III-R criteria and alcoholism defined by DSM-IV criteria showed many, sometimes striking, inconsistencies. These inconsistencies are greatly reduced by making the definition of alcoholism more specific. We defined new phenotypes combining the alcoholism definitions and the latent variables, defining an individual as affected if that individual is alcoholic under one of the definitions (either DSM-III-R or DSM-IV), and indicated having a symptom defined by one of the latent variables. This was done for each of the two alcoholism definitions and five latent variables, selected from a canonical discriminant analyses indicating they formed significant groupings using the electrophysiological variables. We found that linkage analyses utilizing these latent variables were much more robust and consistent than the linkage results based on DSM-III-R or DSM-IV criteria for definition of alcoholism. We also performed linkage analyses on two first prinicipal components derived phenotypes, one derived from the electrophysiolocical variables, and the other derived from the latent variables. A region on chromosome 2 at 250 cM was found to be linked to both of these derived phenotypes. Further examination of the SNPs in this region identified several haplotypes strongly associated with these derived phenotypes.

## Background

An often challenging and sometimes underappreciated facet of genetic epidemiology is the process of choosing an appropriate definition of the trait of interest. Consider for example Alzheimer's disease. While advanced age is the main risk factor for this disease, and it most often strikes after age 65, there are people that develop this disease before the age of 50. Pathologically, the disease appears the same regardless of the age of onset: microscopic examination of brain tissue from patients that are struck with this disease at any age reveal the presence of both amyloid plaques and neurofibrillary tangles. However, it is now recognized that the patients with the very early ages of onset have a mutation in one of three genes (APP, PS1, PS2), and patients that develop Alzheimer's disease at a later age lack these mutations [1,2]. Early-onset Alzheimer's disease is inherited as a Mendelian dominant trait, while the more common form of the disease is a complex trait with a significant environmental component. Thus, any genetic analysis of Alzheimer's disease that did not include age of onset as a component of the disease definition would suffer greatly from the shortcoming known to epidemiologists as misclassification bias. A linkage or association analysis of Alzheimer's disease using only the pathological definition of the disease and ignoring the age of onset would be mixing cases with two distinct genetic etiologies. This could result in a severe loss of power to detect a disease predisposing variant of a gene.

This problem is even more complicated with a behaviorally defined phenotype such as alcoholism, as there is no completely objective laboratory test for the phenotype. In this analysis, we explore the problem of phenotype definition with the Collaborative Study on the Genetics of Alcoholism (COGA) dataset, and look at alternate definitions of the phenotype. Of particular interest is the problem of lack of consistency in results due to a change in the definition of alcoholism from DSM-III-R to DSM-IV criteria [3].

## Methods

We performed model-free linkage analyses using the program SIBPAL, part of the SAGE analysis package [4]. Initial analyses used only microsatellite data. This was later followed up with a combination of microsatellite and Affymetrix single-nucleotide polymorphism (SNP) data using the microsatellite map and the latest SNP map supplied with the data. Data preparation and analyses that did not require accounting for family structure were performed with SAS version 9.0 [5]. This included use of the procedures CANDISC to perform canonical discriminant analysis based on canonical correlation, and PRINCOMP to perform principal components analysis. These techniques were used only as convenient data summary and reduction techniques. Requirements that would make either canonical discriminant analysis or principal component analysis statistically valid (i.e., normality, independence) were not met; however, because we were not directly forming inferences from these results, this is not a major consideration in the current work. SIBPAL performs linkage analysis based on the Haseman-Elston technique [6,7]. Family-based tests of association were performed with the program FBAT [8-10]. Haplotype block structure of the SNPs was explored with the program HAPLOVIEW [11] using the algorithm defined by Gabriel et al. [12].

## Results

### Studies to increase concordance of linkage signals

**Figure 1:**
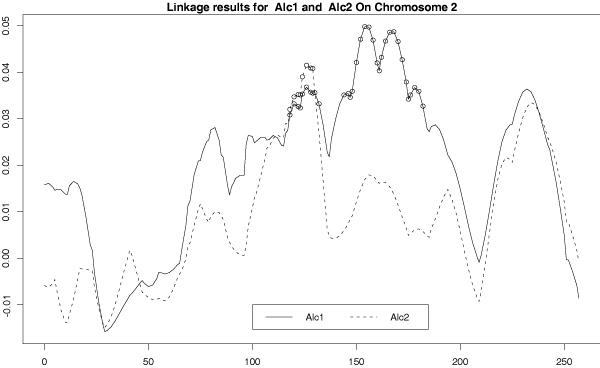
**Initial linkage analysis on chromosome 2**. SIBPAL linkage analysis on chromosome 2 using only STR data. Outcome variables are alcoholism by DSM-III-R criteria (Alc1 – solid line) and alcoholism by DSM-IV criteria (Alc2 – dotted line). Y-axis is the value of the Haseman-Elston regression coefficient. Significant values are indicated with circles.

Two sets of linkage analyses were performed using two different phenotypes: the phenotype of alcoholism defined by each of the two criteria: DSM-IIIR and DSM-IV. These were coded as dichotomous variables, and denoted respectively as Alc1 and Alc2. The following covariates were used in all linkage analyses: dichotomous indicator of habitual smoking; dichotomous indicator of whether an individual was Black (derived from ethnicity); dichotomous indicator of whether an individual was White (derived from ethnicity); age at interview; square of age at interview; cube of age at interview. The results of the initial linkage analyses are shown in Figure 1. The differences in linkage signal when using the two different definitions of alcoholism (Alc1 for DSM-III-R, Alc2 for DSM-IV) are seen throughout the genome.

**Figure 2:**
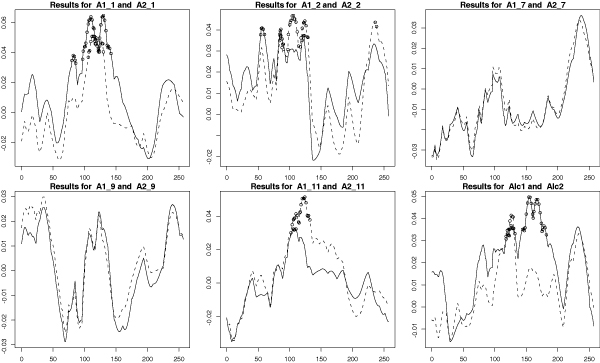
**Linkage analysis of derived phenotypes**. SIBPAL linkage analysis on chromosome 2 using only STR data. Outcome variables are phenotypes derived from Alc1 (DSM-III-R criterion of alcoholism) or Alc2 (DSM-IV criterion of alcoholism) and latent variable 1, 2, 7, 9, or 11. Results from phenotypes derived from Alc1 are graphed as solid lines; those derived from Alc2 are graphed as dotted lines. Y-axis is the value of the Haseman-Elston regression coefficient. Significant values are indicated with circles. The graph from Figure 1 is also included for comparison.

We then made the assumption that the latent variables and electroencephalogram (EEG) variables are phenotypes that could be used to identify more homogeneous alcoholism phenotypes. We performed a canonical discriminant analysis using each of the latent variables as a potential grouping variable for the EEG variables. Although the *p*-values are not valid due to the non-independence of family members, they gave us an approximation of the relationships between the latent variables and the EEG variables. We found that latent variables 1 (persistent desire to stop drinking), 2 (morning drinking), 7 (gave up activities to drink), 9 (withdrawal symptoms), and 11 (emotional psychological problems from drinking) showed evidence of significant grouping with the EEG variables. Motivated by these observations, we defined ten derived phenotypes named Ai_j for i = 1, 2 and j = 1, 2, 7, 9, or 11 as defined above; an individual was affected with derived phenotype Ai_j if they were defined as alcoholic under definition 'i' and also had the symptom defined by latent variable 'j'. Correlations between these phenotypes were all higher than those between the original Alc1 and Alc2 variables (data not shown), and as shown in Figure 2, these phenotypes are much more consistent in their linkage signals than those previously observed using diagnoses based on DSM-IIIR and DSM-IV criteria alone.

**Figure 3:**
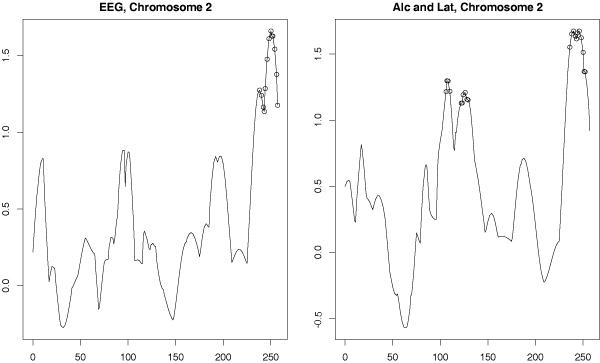
**Linkage analysis of principal component phenotypes**. SIBPAL linkage analysis on chromosome 2 using only STR data. Outcome variables are the first principal components calculated from, respectively: all 13 EEG (electrophysiological) variables; both alcoholism definitions and all latent variables. Y-axis is the value of the Haseman-Elston regression coefficient. Significant values are indicated with circles.

To investigate linkage for phenotypes derived from the EEG variables and the latent variables, we formed several phenotypes using principal components analysis to summarize collections of variables. We used the first principal component derived from all 13 EEG variables, and also the first principal component derived from alcoholism and latent variables. The comparison of the linkage results for these two outcomes is given in Figure 3. There is one region in the neighborhood of 250 cM on chromosome 2 that shows significant linkage with both of the principal component derived phenotypes. While a peak around 100 cM is present in both, it is only significant with the latent variable prinicipal component phenotype.

### SNP fine mapping on chromosome 2q

**Figure 4:**
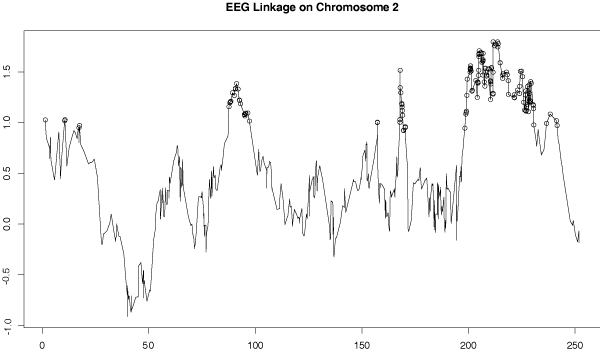
**SNP Linkage analysis of EEG principal component phenotype**. SIBPAL linkage analysis on chromosome 2 using only SNP data. Outcome variable is the first principal component calculated from all 13 EEG (electrophysiological) variables. Y-axis is the value of the Haseman-Elston regression coefficient. Significant values are indicated with circles.

**Figure 5:**
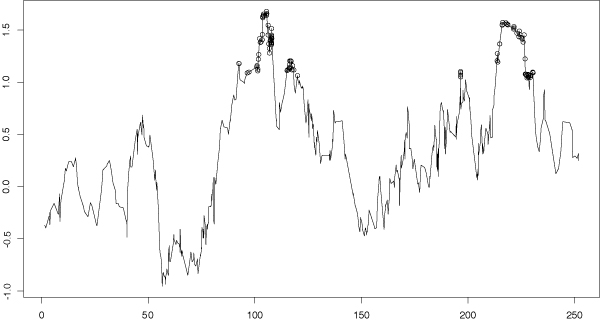
**SNP Linkage analysis of alcoholism and latent variable principal component phenotype**. SIBPAL linkage analysis on chromosome 2 using only SNP data. Outcome variable is the first principal component calculated from both alcoholism definitions and all latent variables. Y-axis is the value of the Haseman-Elston regression coefficient. Significant values are indicated with circles.

We chose to follow up on these chromosome 2 results, especially in the area around 250 cM, with further analyses using the Affymetrix SNP data. We examined the evidence for linkage using only the SNP data for all of chromosome 2. The linkage results for the EEG principal component outcome is shown in Figure 4 and that for latent variable principal component is shown in Figure 5. These results confirm the short tandem repeat (STR) evidence for linkage to the 250-cM region just before the q-terminus for these outcome variables, though the peak signal shifted centromerically to ~220 cM.

**Figure 6:**
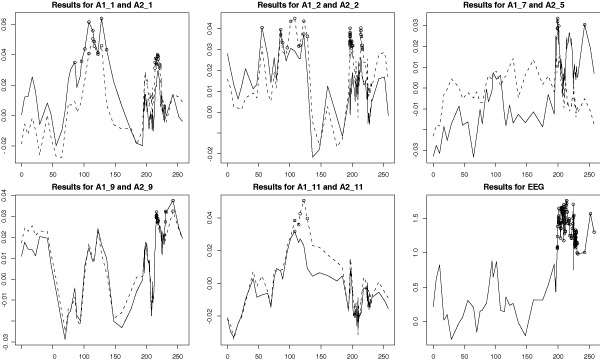
**Combined STR and SNP linkage analysis of derived phenotypes and EEG principal component phenotype**. SIBPAL linkage analysis on chromosome 2 using combined STR and SNP data. Outcome variables for the first five graphs are phenotypes derived from Alc1 (DSM-III-R criterion of alcoholism) or Alc2 (DSM-IV criterion of alcoholism) and latent variable 1, 2, 7, 9, or 11. Results from phenotypes derived from Alc1 are graphed as solid lines; those derived from Alc2 are graphed as dotted lines. Outcome variable for the last graph is the first principal component calculated from all 13 EEG (electrophysiological) variables. Y-axis is the value of the Haseman-Elston regression coefficient. Significant values are indicated with circles.

To attempt to narrow the linkage peaks on 2q, we combined the STR and SNP markers, utilizing a combined map based on the map data sent along with the marker file, which produced the linkage results for our alcohol and latent variable and EEG principal component outcomes shown in Figure 6.

**Figure 7:**
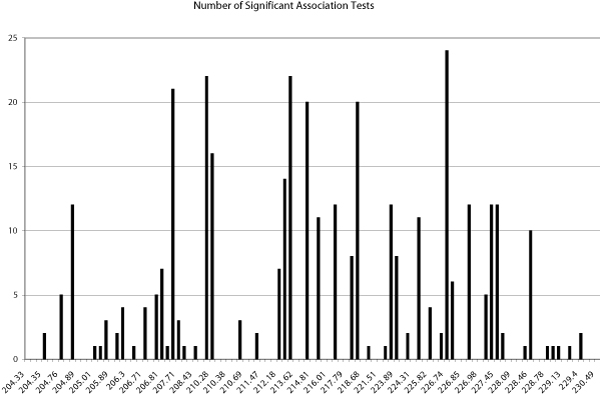
**Number of significant SNP associations by position on chromosome 2**. Of the total 24 possible associations that could be tested for a SNP (see text), the number that were found to be significant (*p *< 0.05) by the FBAT test.

We used the program FBAT to examine evidence for association between SNP genotypes and each of the outcome variables of interest. There were a total of ten phenotypes of type Ai_j, and the two principal component derived phenotypes mentioned above (EEG, and alcoholism/latent). Each association can be tested using either a dominant model for mode of inheritance (i.e., one allele or haplotype dominant over all other alleles or haplotypes), or an additive model (i.e., risk increasing additively with each addition copy of the allele or haplotype). Thus, for a given SNP, up to a total of 24 association tests can be significant. Figure 7 shows the number of significant associations that were found for each SNP in this region by SNP position in centimorgans.

We followed up on these results by examining the associations between these outcome variables and haplotypes formed from SNPs in this region using the HBAT function in the program FBAT. We chose haplotypes based on three criteria: 1) if there were consecutive SNPs that all showed a large number of significant results in Figure 6; 2) if there were consecutive SNPs with identical positions according to the Affymetrix map; and 3) if there were consecutive SNPs determined to be in the same haplotype block by HAPLOVIEW. Using the three criteria, we find the following haplotypes to be significantly associated with most of our phenotypes of interest: using the first we find haplotype tsc0548180|tsc0512074 at positions 209.412–210.28; using the second criteria, we find haplotypes tsc0052569|tsc0530060 at 225.8190 and tsc0977679|tsc0977680 at 226.7420; and finally using the third criteria, we find tsc1282391|tsc0539848 at 206.707–206.751, and tsc0675766|tsc0040071 at 228.372–228.455. Only the first of these (tsc0548180|tsc0512074) was significantly associated with the first EEG principal component. However, there is a 5-SNP haplotype (tsc0045051|tsc0620679|tsc0620681|tsc1612284|tsc0524593; based on the third criterion) at 234.047–235.395 that is significantly associated with this first EEG principal component.

## Discussion

We have identified several alternative phenotypes defined by use of latent and/or EEG variables that provide consistent linkage signals on 2q using STR markers. We confirmed this signal on 2q using the Affymetrix SNP data. We then combined the SNP and STR data and show that the combined information narrows the region and increases the evidence for a candidate region with susceptibility genes for Alcoholism or related traits.

Previous linkage analysis on comorbidity of alcohol dependence and habitual smoking revealed a modest peak on chromosome 2 around 90 cM, while evidence for linkage to our region of interest was weak and inconsistent in that study [13]. Linkage to activity of platelet monoamine oxidase utilizing the COGA dataset was found on chromosome 2 [14]. The region of chromosome 2 in which we found evidence of linkage is consistent with some previous results found for eletrophysiological variables [15] utilizing this same dataset.

Investigating multi-SNP haplotype associations, we find that there are three SNP haplotypes showing consistent and significant associations at positions ~206, 210, and 228 cM on chromosome 2q. However, the principal component derived phenotypes were not found to show significant haplotype associations as those found with the latent variables. However there is a 5-SNP haplotype at 234.0–234.5 that shows significant association with the EEG principal component. We have analyzed multiple phenotypes and multiple SNP haplotypes for these association-based analyses, a total of 24 separate analyses, and have reported here the uncorrected *p*-values. Application of the conservative Bonferroni correction can be easily applied to these *p*-values, though this study is hypothesis generating rather than hypothesis testing, so we felt this unnecessary. Our results mirror the linkage signal previously found utilizing EEG phenotypes reported by Porjesz et al. [15].

## Abbreviations

COGA: Collaborative Study on the Genetics of Alcoholism

EEG: Electroencephalogram

SNP: Single-nucleotide polymorphism

STR: Short tandem repeat

## Authors' contributions

HWW participated in the design of the study and performed the statistical analysis. RCPG conceived of the study design, and participated in the study design and coordination, and helped to draft the manuscript. HT, VG, and GP participated in the selection of appropriate analyses and helped to draft the manuscript. All authors read and approved the final manuscript.
